# Chemotoxicity-induced exosomal lncFERO regulates ferroptosis and stemness in gastric cancer stem cells

**DOI:** 10.1038/s41419-021-04406-z

**Published:** 2021-11-29

**Authors:** Haiyang Zhang, Meng Wang, Yi He, Ting Deng, Rui Liu, Weixue Wang, Kegan Zhu, Ming Bai, Tao Ning, Haiou Yang, Ying Liu, Junyi Wang, Yi Ba

**Affiliations:** Tianjin Medical University Cancer Institute and Hospital, National Clinical Research Center for Cancer, Tianjin’s Clinical Research Center for Cancer, Key Laboratory of Cancer Prevention and Therapy, Tianjin, 300060 China

**Keywords:** Stem-cell research, Gastric cancer

## Abstract

Cancer stem cells (CSCs) are an important cause of tumor recurrence and drug resistance. As a new type of cell death that relies on iron ions and is strictly regulated by intracellular and extracellular signals, the role of ferroptosis in tumor stem cells deserves extensive attention. Mass spectrum was applied to screen for ferroptosis-related proteins in gastric cancer (GC). Sphere-formation assay was used to estimate the stemness of gastric cancer stem cells (GCSCs). Exosomal lnc-ENDOG-1:1 (lncFERO) was isolated by ultracentrifugation. Ferroptosis was induced by erastin and was assessed by detecting lipid ROS, mitochondrial membrane potential, and cell death. Furthermore, a series of functional in vitro and in vivo experiments were conducted to evaluate the effects of lncFERO on regulating ferroptosis and chemosensitivity in GCSCs. Here, we showed that stearoyl-CoA-desaturase (SCD1) played a key role in regulating lipid metabolism and ferroptosis in GCSCs. Importantly, exosomal lncFERO (exo-lncFERO) derived from GC cells was demonstrated to promote SCD1 expression by directly interacting with SCD1 mRNA and recruiting heterogeneous nuclear ribonucleoprotein A1 (hnRNPA1), which resulted in the dysregulation of PUFA levels and the suppression of ferroptosis in GCSCs. Moreover, we found that hnRNPA1 was also involved in lncFERO packing into exosomes in GC cells, and both in vitro and in vivo data suggested that chemotoxicity induced lncFERO secretion from GC cells by upregulating hnRNPA1 expression, leading to enhanced stemness and acquired chemo-resistance. All these data suggest that GC cells derived exo-lncFERO controls GCSC tumorigenic properties through suppressing ferroptosis, and targeting exo-lncFERO/hnRNPA1/SCD1 axis combined with chemotherapy could be a promising CSC-based strategy for the treatment of GC.

## Introduction

Solid tumors, including gastric cancer (GC), usually exhibit intra-tumoral in cell types, cellular composition, intercellular cytokines, matrix proteins, metabolites, and vesicles [1–3]. Cancer stem cells (CSCs), a subpopulation of tumor cells with self-renewal and asymmetrical division properties, are believed to be associated with chemo-resistance and post-treatment tumor relapse in the high mortality of GC patients [4, 5]. Innovative approaches targeting gastric cancer stem cells (GCSCs) self-renewal properties by inducing differentiation or acting on CSC metabolism combined with systemic chemotherapy have led to a new therapeutic option [6, 7].

Ferroptosis is a nonapoptotic, iron-dependent form of regulated cell death occurring when the intracellular levels of lipid reactive oxygen species (lipid ROS) exceed the antioxidant activity of glutathione-dependent peroxidase (GPX4), thus leading to the collapse of cellular redox homeostasis [8, 9]. Recent studies have identified the essential role of ferroptosis in mediating tumor development and drug resistance in various cancers including GC [10–12]. Since aberrant lipid and iron metabolism, along with ROS production, are some of the physiological differences between cancer and normal cells [13, 14], and play essential roles in regulating ferroptosis, cancer cells could be more susceptible to modulation in this death pathway compared to normal cells [15]. Recently, activation of ferroptosis to combat cancer has become a safe and selective therapeutic strategy that has been gaining a lot of attention [16], but the detailed molecular mechanism remains unclear.

Lipid metabolism plays a vital role in various tumor processes, including tumorigenesis, invasion, and metastasis [17]. Since saturated membrane lipids are less sensitive to peroxidation, high levels of saturated membrane lipids protect cancer cells from damage induced by ROS [18, 19], and ferroptosis is driven by lipid peroxidation [20]. It has been established that lipid metabolic pathways were highly upregulated in GCSCs compared with differentiated GC cells, and expression of stearoyl-CoA-desaturase (SCD1) was ranked as the highest [21]. SCD1 catalyzes the synthesis of monounsaturated fatty acids (MUFAs), mainly oleic acid (18:1) and palmitoleic acid (16:1), from their counterparts stearic acid (18:0) and palmitic acid (16:0) [22]. Previous studies have shown that SCD1 plays a key regulatory role in the production of lipid ROS in several types of tumors [23–25]. High levels of phosphatidylethanolamine-containing oxidized polyunsaturated fatty acyl chains (PUFAs), particularly oxidized arachidonate (C20:4) and adrenate (C22:4), have been implicated in ferroptosis [26, 27]. Herein, we hypothesized that SCD1 might affect the sensitivity of GCSCs to ferroptosis.

Exosomes are secreted by most cell types with a typical diameter of 40–100 nm [28]. A large number of studies show that exosomes act as message transmitters in intercellular communication because they deliver a variety of proteins, lipids, long noncoding RNAs (lncRNAs), circRNAs, and microRNAs [29–31]. LncRNAs participate in all stages, including the development, progression, and metastasis of multiple cancers [32–35]. LncRNAs have been demonstrated to be involved in lipid metabolism and ferroptosis [36–39].

In this study, exosomal lnc-ENDOG-1:1 was found to be linked with lipid ROS production on the dynamic regulation of ferroptosis in GCSCs, and was named exosomal lncFERO (exo-lncFERO). Data from both in vitro and in vivo experiments indicated that GC-secreted exo-lncFERO upregulated the expression of SCD1 in GCSCs by binding with SCD1 mRNA and recruiting heterogeneous nuclear ribonucleoprotein A1 (hnRNPA1), leading to suppressed ferroptosis in GCSCs and the decreased chemosensitivity of gastric tumors. Therefore, our study illustrated a novel pathway comprising exosomes, lncRNA, hnRNPA1, and SCD1 between GC and CSC, and suggested that targeting ferroptosis-related genes in CSC serves as a new method for clinical treatment of solid tumors.

## Results

### SCD1 is closely related to lipid metabolism and ferroptosis in GC

**Fig. 1:**
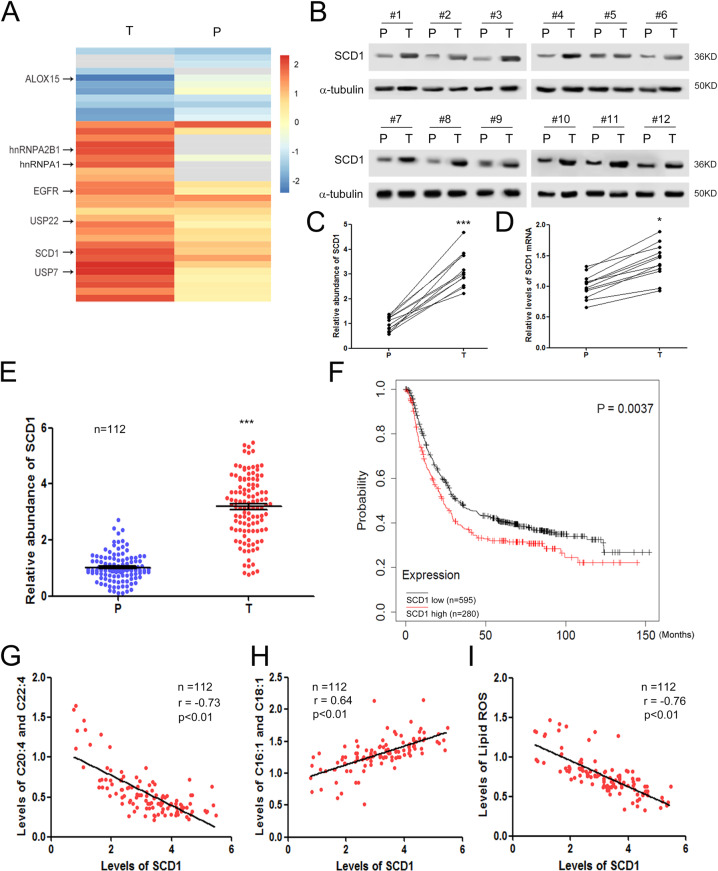
SCD1 is related to lipid metabolism and ferroptosis in gastric cancer. **A** Mass spectrum analysis of protein expression changes in gastric tumor tissues (*n* = 45). The heatmap depicts the relative protein abundance in tumor tissues (T) and paired paracarcinoma tissues (P). **B** Validation of SCD1 dysregulation in GC by using western blotting analysis (*n* = 12). **C** Quantitative analysis of (**B**) (*n* = 12). **D** Relative levels of SCD1 mRNA in gastric tumor tissues (*n* = 12). **E** Validation of SCD1 dysregulation in GC tissues using protein microarray (*n* = 112). **F** High expression of SCD1 predicts poor overall survival in GC. Patients were divided into the SCD1 high group (*n* = 280) and SCD1 low group (*n* = 595) based on the average value of SCD1 mRNA levels. **G** SCD1 is negatively correlated with polyunsaturated fatty acids (PUFAs) (C20:4 and C22:4) (*n* = 112). **H** SCD1 is positively correlated with monounsaturated fatty acids (MUFAs) (C16:1 and C18:1) (*n* = 112). **I** SCD1 is negatively correlated with lipid ROS production (*n* = 112). *p < 0.05 and ***p < 0.001.

SCD1 has been reported to play a key regulatory role in the production of lipid ROS in various types of tumors according to previous studies [23–25]. First, we screened GC-specific proteins by using mass spectrometry (*n* = 45). The results showed that a group of proteins was significantly dysregulated in GC tumor tissues (T) compared with paracarcinoma tissues (P), and SCD1 were clearly increased in tumor tissues (Fig. 1A). The levels of SCD1 protein were validated by western blotting (WB) analysis and the levels of SCD1 mRNA were measured by qRT-PCR. It was shown that SCD1 protein was upregulated more than threefold (Fig. 1B, C), but SCD1 mRNA only showed slight increase in tumor tissues (Fig. 1D). Consistent with these results, SCD1 was significantly increased in tumor tissues compared with paracarcinoma tissues (*n* = 112) (Fig. 1E). Subsequently, we divided GC patients into the SCD1 high group (*n* = 280) and the SCD1 low group (*n* = 595) based on the mean value of SCD1 expression. We found that high levels of SCD1 predicted poor overall survival (OS) in GC (Fig. 1F), indicating that SCD1 acts as a cancer-promoting factor in GC. We focused on the biochemical properties of SCD1 and its known effect on lipid metabolism from previous studies [40]. In addition, lipids have been linked to lipid ROS production [41], and oxidized PUFAs mediate ferroptosis [26]. Our data showed that in 112 GC patients, SCD1 was negatively correlated with PUFA levels (Fig. 1G), but positively correlated with MUFA levels (Fig. 1H), and SCD1 was negatively correlated with lipid ROS production (Fig. 1I). These data verified that SCD1 plays a key role in mediating lipid metabolism and ferroptosis in gastric tumors.

### Exosomal lncFERO shows clinic correlation with SCD1

**Fig. 2:**
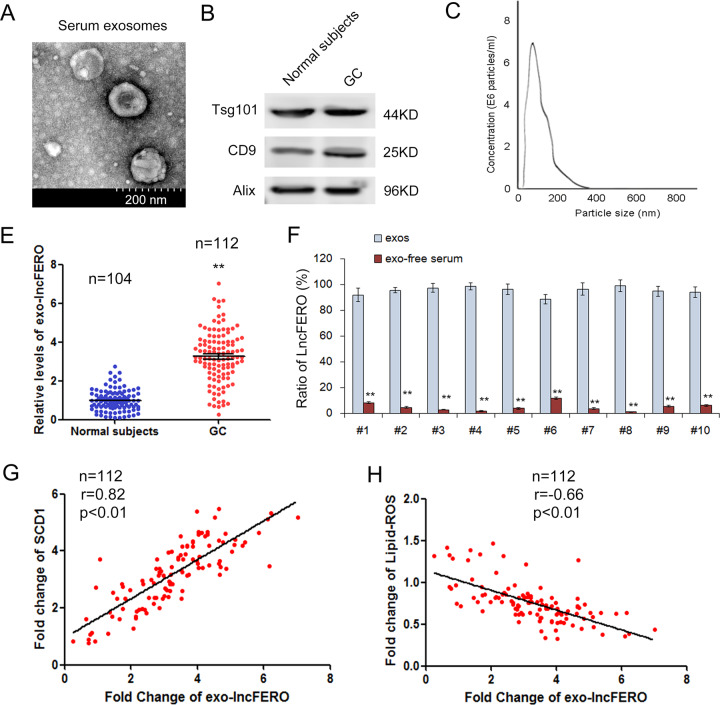
Exosomal lncFERO is positively linked with SCD1 expression. **A** Serum exosomes were observed by using an electron microscope (scale bar, 200 nm). **B** Detection of exosome markers, Tsg101, CD9, and Alix, by western blotting assay. **C** Nanotracking analysis of the diameters of vesicles. **E** Relative levels of exo-lncFERO in normal subjects (*n* = 104) and GC patients (*n* = 112). **F** Relative levels of exo-lncFERO in the exos and exo-free serum (*n* = 10). **G** Exo-lncFERO is positively correlated with SCD1 (*n* = 112). **H** Exo-lncFERO is negatively correlated with lipid ROS levels (*n* = 112). **p < 0.01.

To screen out the specific exosomal lncRNA that was involved in SCD1 expression, serum exosomes were isolated from both normal subjects and GC patients and were used for subsequent lncRNA microarray. The images of serum exosomes were obtained by electron microscopy (Fig. 2A), and the exosome markers, Tsg101, CD9, and Alix were detected by WB (Fig. 2B). Consistent with the typical size of exosomes, the diameters of vesicles were in the range 30–150 nm, as demonstrated by nanotracking analysis (Fig. 2C). It has been reported that lncRNAs play critical roles in the development, progression, and metastasis of multiple cancers [42, 43]. The expression profile of exosomal lncRNAs was determined by microarray, and the top 20 upregulated lncRNAs in GC are listed in Supplementary Table 1. The level of lnc-ENDOG-1:1, named lncFERO (ferroptosis-associated lncRNA) in our study, ranked third in GC exosomes. Further qRT-PCR analysis verified that serum exosomal lncFERO was significantly increased in GC patients (*n* = 112) compared with healthy subjects (*n* = 104) (Fig. 2E). To determine the form of lncFERO’s presence in serum, the levels of lncFERO in isolated exosomes and exosome-free serum were detected respectively, and the data clearly showed that more than 90% serum lncFERO exist in exosomes (Fig. 2F). Moreover, we also found that exo-lncFERO was positively correlated with SCD1 expression (Fig. 2G), but negatively linked with lipid ROS production (Fig. 2H). These results suggested that there is potential clinical relevance among lncFERO, SCD1, and lipid ROS accumulation.

### The expression of lncFERO is decreased in GCSCs compared with GC cells

**Fig. 3:**
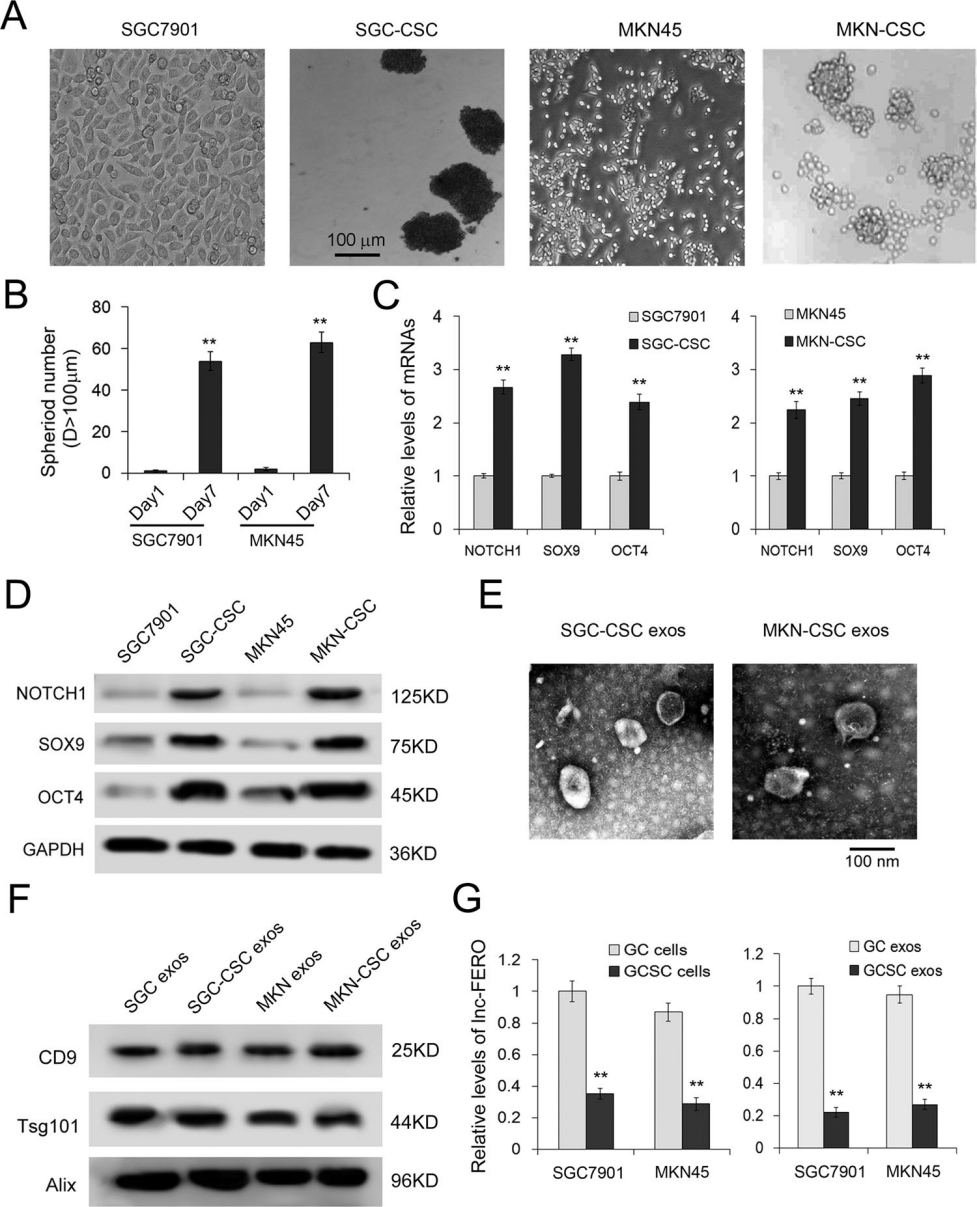
The different expression characters of lncFERO in GC cells and GC stem cells. **A** Sphere-formation assays were performed in indicated cell lines. **B** Number of tumorspheres (*n* = 3). **C** qRT-PCR analysis of stemness-associated genes, including NOTCH1, SOX9, and OCT4, in GC and GCSC cell lines (*n* = 3). **D** Detection of stemness-associated genes by western blotting assay (*n* = 3). **E** Exosomes images obtained from GCSCs were observed by using an electron microscope (scale bar, 100 nm). **F** Detection of exosome markers, CD9, Tsg101, and Alix, by western blotting assay. **G** Relative levels of lncFERO in GC cells and GCSCs (*n* = 3). **p < 0.01.

Known as the primary cause of invasiveness, drug resistance, and metastasis [44], CSCs have been identified in GC [45, 46]. In this study, CSCs were obtained from GC cell lines by using FBS-free medium and nonadherent plates. The images of spheres formed by GCSCs are shown in Fig. 3A, B. We detected the expression of stemness-associated genes, including NOTCH1, SOX9, and OCT4. The results showed that both mRNA and protein levels were significantly increased in GCSCs compared with GC cells (Fig. 3C, D). Then, we used ultracentrifugation to isolate exosomes from GCSCs and observed the vesicles by electron microscopy (Fig. 3E). All the exosome markers tested, Tsg101, CD9, and Alix, were detected in both GC and GCSC exosomes by WB assay (Fig. 3F). As expected, the levels of lncFERO were also downregulated in both GCSCs and GCSC exosomes compared with GC groups (Fig. 3G). These data illustrated that lncFERO is mainly secreted from GC, and may act on GCSC in tumor microenvironment.

### Exosomal lncFERO derived from GC cells inhibits ferroptosis in GCSCs

**Fig. 4:**
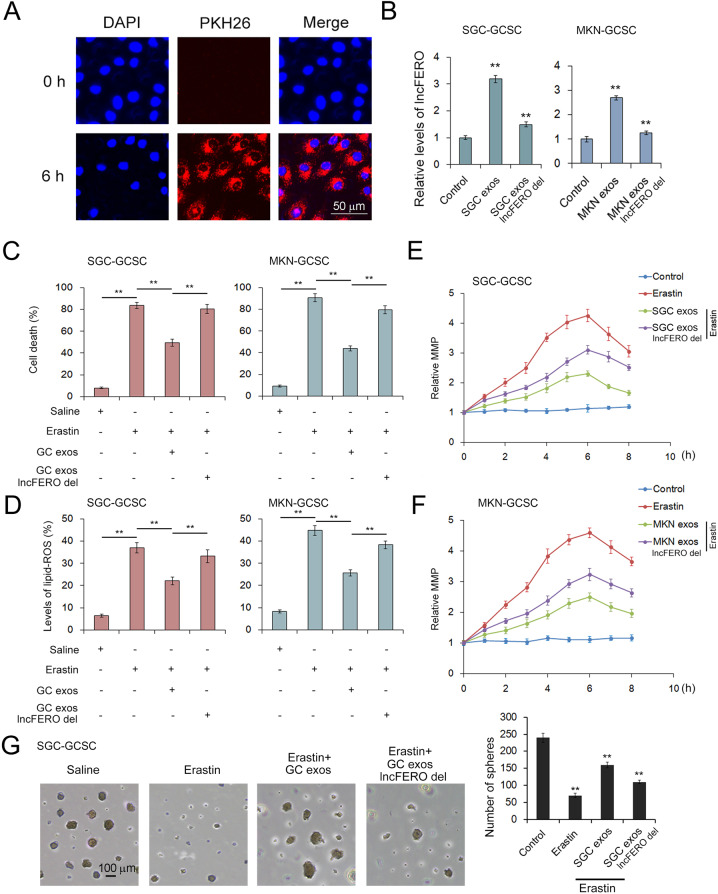
Exosome-delivered lncFERO suppresses ferroptosis in GCSCs. **A** Confocal microscopy image of the internalization of fluorescently labeled GC exosomes in GCSCs. **B** Effects of GC exosomes on lncFERO levels in GCSCs (*n* = 3). **C**–**F** Exo-lncFERO derived from GC cells suppresses erastin-induced ferroptosis in GCSCs. GC exo-lncFERO decreases erastin-induced cell death (**C**) (*n* = 3), inhibits lipid ROS accumulation (**D**) (*n* = 3), and reduces abnormal increases in MMP (**E**, **F**) (*n* = 3). **G** Effects of erastin and exo-lnc-FERO on sphere formation of GCSCs (*n* = 3). **p < 0.01.

To test the function of lncFERO derived from GC cells in the regulation of ferroptosis in GCSCs, GC exosomes were isolated and cocultured with GCSCs, and PKH26-labeled GC exosomes were detected in GCSCs at 6 h (Fig. 4A), which showed that exosomes derived from GC cells can fuse with GCSCs efficiently. The levels of lncFERO in GCSCs cocultured with GC exosomes were detected by using qRT-PCR. The highest level of lncFERO was found in the GC exosome group, followed by the GC exosomal lncFERO deletion group (Fig. 4B). Subsequently, exo-lncFERO was proven to dramatically suppress erastin-induced ferroptosis (Fig. 3C), lipid ROS accumulation (Fig. 4D) in GCSCs, and abnormal mitochondrial membrane potential (MMP) increase (Fig. 4E, F). Erastin also decreased the sphere-formation ability of GCSCs, which was partly rescued by exo-lncFERO (Fig. 4G). Thus, GC-derived exo-lncFERO inhibits ferroptosis and enhances stemness of GCSCs.

### Exo-lncFERO secreted from GC cells recruits hnRNPA1 and promotes SCD1 expression in GCSCs

**Fig. 5:**
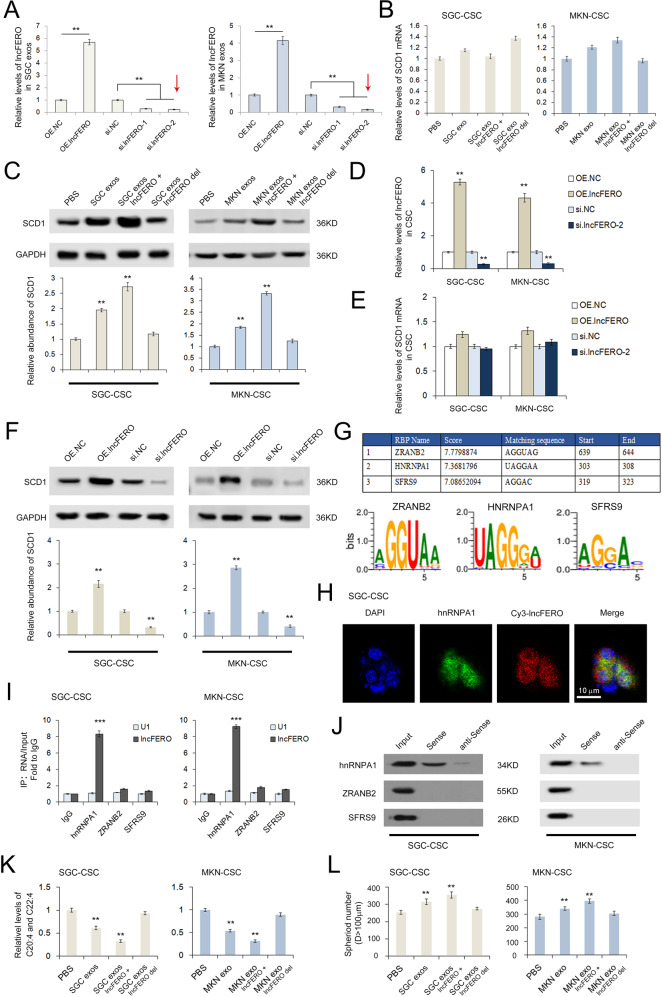
LncFERO promotes SCD1 expression by recruiting hnRNPA1 in GCSCs. **A** Levels of lncFERO in exosomes isolated from GC cells treated with lncFERO-OE plasmids or siRNAs (*n* = 3). **B** Effects of GC-secreted lncFERO on SCD1 mRNA levels in GCSCs (*n* = 3). **C** Effects of GC-secreted lncFERO on SCD1 expression in GCSCs (*n* = 3). **D** lncFERO in GCSCs was overexpressed or knocked down (*n* = 3). **E** Effects of lncFERO on the expression of SCD1 mRNA in GCSCs (*n* = 3). **F** Western blotting analysis of SCD1 expression in GCSCs with overexpression or knockdown of lncFERO (*n* = 3). **G** RBPDB analysis of the specific interaction between lncFERO and RBP motifs. **H** The colocalization of Cy3-lncFERO and hnRNPA1 in GCSCs (*n* = 3). **I** RNA immunoprecipitation assay shows the direct interaction between lncFERO and hnRNPA1 in GCSCs (*n* = 3). **J** Detection of hnRNPA1 protein in the samples derived from lncFERO pulldowns performed in GCSCs. **K** GC exosomal lncFERO decreased PUFA levels in GCSCs (*n* = 3). **L** GC exosomal lncFERO promoted the stemness of GCSCs (*n* = 3). **p < 0.01 and ***p < 0.001.

Previous studies have shown that SCD1 may be involved in one or more complex pathways linked to tumor ferroptosis [25, 47]. Although we have proven that exo-lncFERO has a positive correlation with SCD1, the specific interaction remains unclear. To determine the direct interaction between lncFERO and SCD1, SGC7901 and MKN45 cells were treated with lncFERO-OE plasmids or siRNAs, and the levels of lncFERO in exosomes are shown in Fig. 5A, and si.lncFERO-2 (marked by red arrow) was selected for the subsequent experiments. Exo-lncFERO isolated from GC cells had little effect on SCD1 mRNA (Fig. 5B) but clearly promoted SCD1 protein expression in GCSCs (Fig. 5C). Subsequently, we treated GCSCs in the same manner by using lncFERO-OE plasmids or siRNAs and observed the same results (Fig. 5D–F). Thus, it was concluded that lncFERO positively regulated SCD1 expression at the posttranscriptional level.

The potential lncFERO binding proteins were predicted by using RBPDB (the database of RBP specificities, http://rbpdb.ccbr.utoronto.ca/). The top three RNA binding proteins, ZRANB2, hnRNPA1, and SFRS9, are listed in Fig. 5G. Cy3-labeled lncFERO was found to be colocalized with hnRNPA1 in GCSCs (Fig. 5H). Moreover, RNA immunoprecipitation (RIP) assay showed that lncFERO was only detected in the hnRNPA1 group (Fig. 5I), and hnRNPA1 was also detected in the RNA pulldown assay using lncFERO sense probes (Fig. 5J). As expected, GC exosomal lncFERO decreased PUFA levels in GCSCs (Fig. 5K) and enhanced the spheroid-forming efficiency of GCSCs (Fig. 5L).

In summary, these data showed that GC-secreted lncFERO promotes SCD1 expression by recruiting hnRNPA1 and thus regulates PUFA levels and stemness in GCSCs.

### LncFERO binds to the 5’UTR of SCD1 mRNA and promotes SCD1 translation by recruiting hnRNPA1

**Fig. 6:**
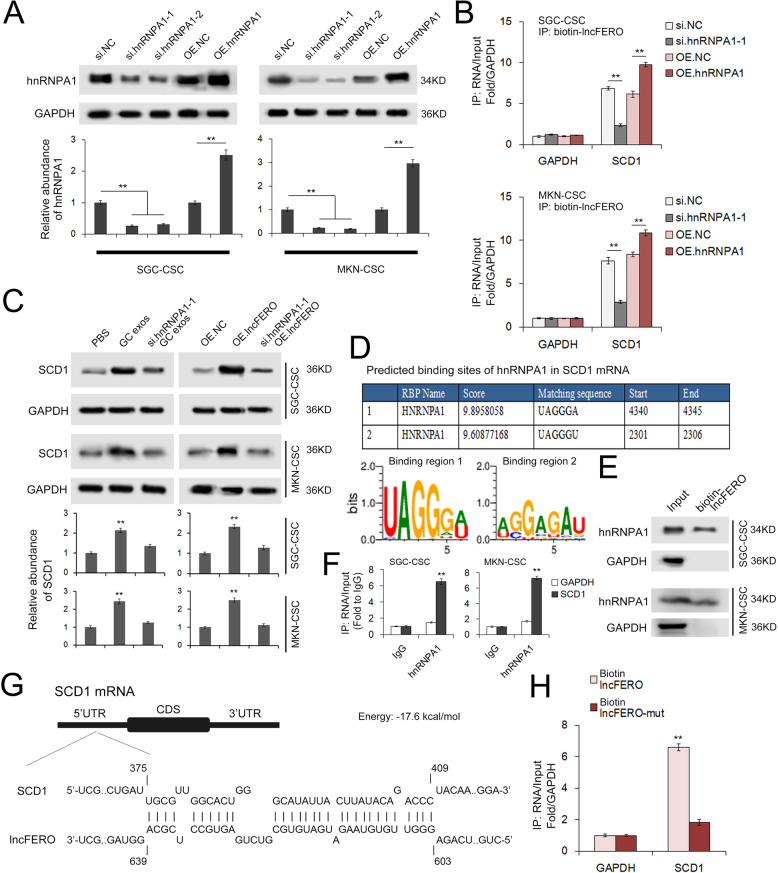
LncFERO interacts with SCD1 mRNA and promotes SCD1 translation by recruiting hnRNPA1. **A** hnRNPA1 in GCSCs was overexpressed or knocked down (*n* = 3). **B** hnRNPA1 stabilizes the interaction between lncFERO and SCD1 mRNA (*n* = 3). **C** GC exosomal lncFERO depends on hnRNPA1 to promote SCD1 expression in GCSCs (*n* = 3). **D** RBPDB analysis of the specific interaction between hnRNPA1 and SCD1. **E** Detection of hnRNPA1 protein in the samples derived from lncFERO pulldowns performed in GCSCs (*n* = 3). **F** RIP shows the direct interaction between SCD1 mRNA and hnRNPA1 in GCSCs (*n* = 3). **G** Predicted binding sites of lncFERO in the 5’UTR of SCD1 mRNA. **H** Capture of SCD1 mRNA by biotin-labeled lncFERO (*n* = 3). **p < 0.01.

It has been reported that the hnRNP family is required for packaging a number of noncoding RNAs and mRNAs into exosomes [48, 49]. In addition, hnRNPA1 has been shown to be upregulated in gastric tumor tissues by MS. To further investigate the inner link between SCD1, hnRNPA1, and lncFERO, we constructed plasmids containing hnRNPA1 coding sequences, as well as two siRNAs (si.hnRNPA1-1 and si.hnRNPA1-2). Transfections of both siRNAs, especially si.hnRNPA1-1, led to the reduction of hnRNPA1, while the plasmids obviously upregulated hnRNPA1 expression in GCSCs (Fig. 6A). RNA pulldown assay using biotin-labeled lncFERO further validated the interaction between lncFERO and SCD1 mRNA in GCSC, which was found to be dependent on hnRNPA1 (Fig. 6B). The knockdown of hnRNPA1 in GCSCs blocked the effects of SGC exosomes and lncFERO on SCD1 expression (Fig. 6C). Next, we used RBPDB to predict the potential binding sites of hnRNPA1 and SCD1 mRNA. The two binding regions are shown in Fig. 6D. We detected hnRNPA1 expression in the samples derived from lncFERO pulldowns (Fig. 6E). Moreover, SCD1 mRNA, instead of GAPDH mRNA, could be detected in the product of RIP by using the anti-hnRNPA1 antibody (Fig. 6F). Bioinformatics analysis showed that lncFERO interacts with the 5’UTR of SCD1 mRNA (Fig. 6G). Furthermore, SCD mRNA was clearly detected in the RNA pulldown product by using biotin-labeled wild-type lncFERO but not with mutated lncFERO (Fig. 6H). Collectively, these data provided direct evidence to show that lncFERO binds to SCD1 mRNA to promote SCD1 translation in an hnRNPA1-dependent manner.

### Cisplatin and paclitaxel promotes lncFERO secretion through USP7/hnRNPA1 axis

**Fig. 7:**
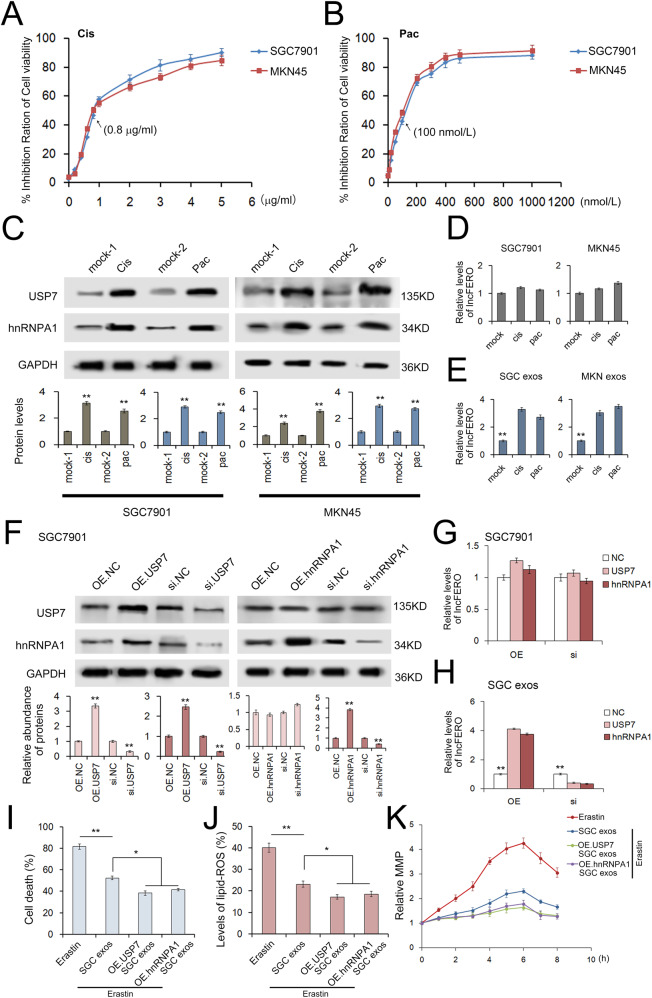
Chemotoxicity promotes lncFERO secretion from GC cells via the USP7/hnRNPA1 axis. **A**, **B** The effects of gradient doses of cisplatin (**A**) and paclitaxel (**B**) on the viability of SGC7901 and MKN45 cells (*n* = 3). **C** The expression of USP7 and hnRNPA1 in GC cells treated with sublethal doses of cisplatin (0.8 μg/mL) and paclitaxel (100 nmol/L) (*n* = 3). **D** Effects of cisplatin and paclitaxel on the expression of lncFERO in GC cells (*n* = 3). **E** Effects of cisplatin and paclitaxel on the secretion of lncFERO in GC exos (*n* = 3). **F** WB analysis of USP7 and hnRNPA1 in SGC7901 cells treated with corresponding overexpression plasmids or siRNAs (*n* = 3). **G** Effects of USP7 and hnRNPA1 on the expression of lncFERO in SGC7901 cells (*n* = 3). **H** Effects of USP7 and hnRNPA1 on the expression of lncFERO in SGC exos (*n* = 3). **I**–**K** Exosomes derived from SGC7901 cells overexpressing USP7 and hnRNPA1 suppressed erastin-induced cell death (**I**), lipid ROS production (**J**), and the MMP increase (**K**) in GCSCs (*n* = 3). *p < 0.05 and **p < 0.01.

To analyze the chemotherapy-induced damage responses in GC cells, we treated cells with gradient doses of cisplatin or paclitaxel, and 0.8 μg/mL cisplatin and 100 nmol/L paclitaxel were selected as the sublethal doses for the following experiments (Fig. 7A, B). Herein, the results showed that both cisplatin and paclitaxel promoted USP7 and hnRNPA1 expression in GC cells (Fig. 7C), which resulted in the upregulation of lncFERO in exosomes without changing lncFERO expression in GC cells (Fig. 7D, E). Overexpressed USP7 relatively increased hnRNPA1 levels, while the knockdown of USP7 decreased hnRNPA1 expression (Fig. 7F). In addition, the overexpression of either USP7 or hnRNPA1 promoted lncFERO in exosomes without obviously changing lncFERO expression in GC cells, while the knockdown of both genes showed the opposite effects (Fig. 7G, H). By using an immune-precipitation assay, USP7 could be detected in the product of the anti-hnRNPA1 antibody, and vice versa (Supplementary Fig. 1A). In addition, the data also showed a negative link between USP7 and the levels of hnRNPA1 ubiquitination (Supplementary Fig. 1B).

Moreover, exosomes isolated from SGC7901 cells overexpressing either USP7 or hnRNPA1 showed a stronger ability to inhibit erastin-induced cell death, lipid ROS production, and abnormal MMP increase (Fig. 7I–K). We also checked the effects of USP7 and hnRNPA1 on biological behavior of cancer cells, both USP7 and hnRNPA1 showed a positive link with increased cell viability (Supplementary Fig. 2A, B), but had only slight influence on cell cycle (Supplementary Fig. 3A, B).

Collectively, these results implied that chemotoxicity promotes lncFERO packaging into exosomes by increasing the expression of USP7, which stabilizes hnRNPA1 in GC cells via deubiquitination.

### Validation of the exo-lncFERO’s role in regulating ferroptosis, stemness, and chemosensitivity by using tumor-implanted mice

**Fig. 8:**
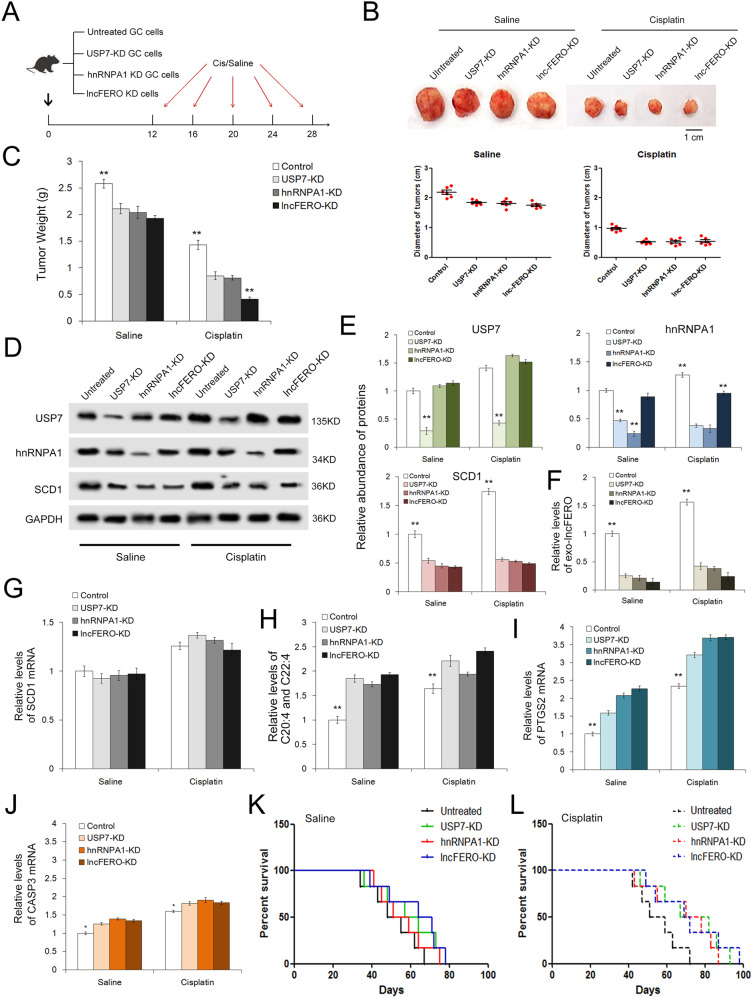
In vivo role of the USP7/hnRNPA1/lncFERO pathway in regulating ferroptosis and chemosensitivity of gastric tumors. **A** Schematic description of the experimental design used to establish the animal model. **B** Images and the diameters of tumors in each group (*n* = 6). **C** Weight measurements of the tumors described above (*n* = 6). **D** WB analysis of USP7, hnRNPA1 and SCD1 in tumor tissues (*n* = 3). **E** Quantitative analysis of (**D**) (*n* = 3). **F** Relative levels of serum exo-lncFERO in each group (*n* = 6). **G** Relative levels of SCD1 mRNA in tumor tissues (*n* = 6). **H** Relative levels of PUFAs in tumor tissues (*n* = 6). **I**, **J** Quantification of a ferroptosis marker (**I**) and an apoptosis marker (**J**) in each group (*n* = 6). **K**, **L** Kaplan–Meier curves of mice in the saline-treated groups (**K**) and cisplatin-treated groups (**L**) (*n* = 6). **p < 0.01.

Finally, we evaluated the function of the secreted lncFERO in affecting tumor growth and chemotherapeutic efficacy in vivo. Three stable cell strains with knockdown of USP7, hnRNPA1, or lncFERO were constructed by using lent-viruses containing shRNAs, and then the cell strains were used for subcutaneous tumor implantation (Fig. 8A). The tumor-implanted mice were injected via the lateral tail vein with either cisplatin (5 μg/g) or saline every 4 days starting on day 12, and the treatment was completed on day 28. The knockdown of USP7, hnRNPA1, or lncFERO in GC cells relatively suppressed tumor growth in the saline groups, but remarkably increased chemosensitivity in the cisplatin groups (Fig. 8B, C). WB analysis showed that knockdown of USP7 suppressed hnRNPA1 expression, while the knockdown of each of the three genes led to a decrease in SCD1 protein, and cisplatin slightly improved the levels of USP7, hnRNPA1, and SCD1 compared with those in the saline groups (Fig. 8D, E). The knockdown of USP7/hnRNPA1/lncFERO also caused a sharp decrease in exosome lncFERO in mouse serum (Fig. 8F). However, little change was observed in SCD1 mRNA (Fig. 8G). The levels of PUFAs showed an increase in the USP7/hnRNPA1/lncFERO-KD groups and were the highest in the lncFERO-KD group (Fig. 8H). Cisplatin was found to promote the expression of stemness-associated genes, including NOTCH1, SOX9, and OCT4, which were inhibited with the knockdown of USP7/hnRNPA1/lncFERO (Supplementary Fig. 4). Subsequently, we detected the changes in the ferroptosis marker PTGS2 and the apoptosis marker CASP3. PTGS2 was clearly upregulated, while CASP3 showed little only increase in USP7-KD, hnRNPA1-KD, and lncFERO-KD cells (Fig. 8I, J). Longer OS was observed in tumor-bearing mice transplanted with USP7-KD, hnRNPA1-KD, and lncFERO-KD cells (Fig. 8K). In addition, the mice treated with cisplatin showed a better survival than those treated with saline (Fig. 8L). In conclusion, these in vivo data showed that USP7/hnRNPA1 facilitates exo-lncFERO secretion from GC, inhibits ferroptosis, enhances stemness, and regulates chemosensitivity in vivo.

### A model illustrating the role of GC-derived exosomal lncFERO in regulating ferroptosis in GCSC

We provide the following schematic diagram that explains the biological function of the transcellular signaling pathway, including USP7, hnRNPA1, exo-lncFERO, and SCD1, in regulating ferroptosis of GCSC (Supplementary Fig. 5).

## Discussion

CSC has been known to play an important role in treatment failure, chemo-resistance, and recurrence in different types of tumors. The interaction between CSC and others cells in tumor microenvironment is important for stemness maintenance and cancer progression [50]. Extracellular vesicles have been reported to promote breast cancer stemness [51]. Our study focused on the crosstalk between GC cells and GCSCs.

Ferroptosis is an oxidative, iron-dependent form of cell death that is related to cancer suppression by the accumulation of lipid peroxidation products [9, 52, 53]. Recent studies have indicated that ferroptosis may act as a tumor suppressive mechanism downstream of p53 [10, 54]. Some researchers have shown that using small molecule activators of ferroptosis might selectively eliminate cancer cells with mutations in the RAS-RAF-MEK pathway, but the conclusion remains controversial [55–57]. Therefore, the mechanism of how the ferroptosis pathway is regulated remains unclear. Lipids participate in complex ways in cell death pathways, acting as both initiators and facilitators of apoptosis and impacting necroptosis and ferroptosis. Lipid oxidation appears to be particularly central to the process of ferroptosis. SCD1 may be regulated through more than one pathway that is associated with tumor suppressors, and genetic or pharmacologic blockade of SCD1 induces ferroptosis as well as apoptosis [24]. However, the mechanism by which SCD1 acts on GC remains poorly understood. Although lncRNAs, as important molecules, play regulatory roles in multiple physiological and pathological processes in the cell, their role in the regulation of oxidative stress has not been clearly described.

For the treatment of GC, chemotherapy is still the main method for advanced cancers. As first-line chemotherapeutic drugs, cisplatin and paclitaxel are widely used in the clinic; however, resistance to cisplatin and paclitaxel has become increasingly common. In general, chemotherapy resistance is related to DNA damage repair, mutations of the molecules regulating cell apoptosis and increased levels of glutathione [58]. It is worth exploring whether we can solve the problem of chemotherapy resistance through the ferroptosis-related signaling pathway.

In this study, we identified a novel network mediated by exosomes that regulates ferroptosis and stemness of GCSC, in which exo-lncFERO derived from GC cells regulates SCD1 protein expression with the assistance of hnRNPA1, resulting in decreased content of polyunsaturated fatty acids, suppressed ferroptosis, and advanced stemness in GCSC. And USP7 promotes the packaging of lncFERO into exosomes from GC cells through regulating deubiquitination on hnRNPA1, which ultimately leads to chemo-resistance and tumor progression through the exo-lncFERO/hnRNPA1/SCD1 axis (Supplementary Fig. 5).

Our study suggested that targeting the exosome-mediated crosstalk between CSCs and cancer cells serves as an efficient method for the prevention of chemo-resistance as well as gastric tumor recurrence.

## Materials and methods

### Human tissues

All the tumor tissue samples and plasma samples of GC patients were obtained from Tianjin Medical University Cancer Institute and Hospital. Our study was approved by the Ethics Committee of Tianjin Medical University Cancer Institute and Hospital. Informed consent was obtained from the patient to publish this study. All the study methodologies conformed to the standards set by the Declaration of Helsinki.

### Cell lines and culture

Human gastric cancer cell lines (SGC7901 and MKN45) were purchased from the cell bank of the Chinese Academy of Sciences (Shanghai, China). Cells were frozen at low passage and used within 2–3 months after thawing. SGC7901 cells and MKN45 cells were cultured in DMEM (Gibco, USA) and RPMI-1640 (Gibco, USA), supplemented with 10% FBS, respectively. Human gastric cancer stem cell lines (SGC-CSC and MKN-CSC) were isolated from SGC7901 and MKN45 cells by performing a sphere-forming culture. In short, the GC cells were plated at a density of 10^4^ cells/well in ultra-low-attachment 6-well plates (Corning) with stem cell medium, comprised GlutaMAX-DMEM/F12 (Gibco, USA), 2% B27 (Invitrogen), epidermal growth factor (20 ng/mL, Peprotech), basic fibroblast growth factor (10 ng/mL, Peprotech), 0.4% bovine serum albumin (Solarbio), and 5 μg/mL insulin (Novo Nordisk). Culture medium was supplemented with additional growth factors every 3 days, and the cells were maintained in stem cell medium for 7 days. Tumorspheres were collected, dissociated into single-cell suspensions, and cultured to acquire the regeneration of tumorspheres. Second-passage tumorspheres were used for all relevant experiments. All cells were maintained at 37 °C in a 5% CO_2_ humidified atmosphere and tested for mycoplasma contamination before use.

### Sphere-formation assay

The tumorsphere formation assay was performed as previously described to explore the self-renewal capacity [21, 59]. The cells were inoculated in ultra-low-attachment 6-well plates (Corning) at an appropriate density with the stem cell medium described above. Each well was checked every 3 days by light microscopy, The quantity of tumorspheres of 100 μm or more was counted 7 days later. Images of tumorspheres were taken using a ZOE™ Fluorescent Cell Imager (Bio-Rad, CA), and tumor size was measured using ImageJ software.

### Animals

Nude mice (BALB/c-nu, 6–8 weeks old) were purchased from the Model Animal Center of Nanjing University and housed in a specific pathogen-free animal facility. All the experimental procedures were approved by the Institutional Animal Care and Research Advisory Committee of Tianjin Medical University Cancer Institute and Hospital.

### Isolation of exosomes

According to previous reports [60], exosomes in medium and in serum were isolated by differential centrifugation. First, cells and other debris were removed by centrifugation at 300 × g and 3000 × g, respectively, and then, the large-sized shedding vesicles were removed by centrifugation at 10,000 × g for 30 min from the supernatant. Finally, exosomes were contained in the pellet by centrifugation at 100,000 × g for 70 min from the supernatant and suspended in PBS. All steps above were performed at 4 °C.

### PKH26 staining

The PKH26 Red Fluorescent Cell Linker Kit (Sigma) was utilized for exosome staining. Fifty microgram of exosomes (quantified by mass concentration with NanoDrop 2000 Spectrophotometer, Thermo, Waltham, MA, USA) resuspended in 100 μL of diluent C was mixed with 100 μL PKH26 dye solution (4 × 10^−6^ M) and incubated for 1–5 min, which was stopped by adding 200 μL of serum. The labeled exosomes were then washed twice with PBS and coincubated with recipient cells in one well of a dish for 2–24 h before imaging was performed.

### Transmission electron microscopy (TEM) assay

Exosomes were fixed in 2.5% glutaraldehyde at pH 7.2 at 4 °C overnight. The samples were washed in PBS buffer three times (10 min each time) and then fixed in 1% osmium tetroxide at room temperature for 60 min. The samples were prepared as follows: the samples were embedded in 10% gelatin, fixed in glutaraldehyde at 4 °C, and cut into several blocks (less than 1 mm). Then, dehydration of the samples was performed in increasing concentrations of alcohol (30%, 50%, 70%, 90%, 95%, and 100% × 3, 10 min, each step). After that, infiltration of the samples was performed with increasing concentrations of Quetol-812 epoxy resin mixed with propylene oxide (25%, 50%, 75%, and 100%, 3 h, each step). Finally, the samples were embedded in pure, fresh Quetol-812 epoxy resin and polymerized at 35 °C for 12 h, 45 °C for 12 h, and 60 °C for 24 h. Ultrathin sections (100 nm) were cut using a Leica UC6 ultramicrotome and then stained with uranyl acetate for 10 min and lead citrate for 5 min at room temperature. An FEI Tecnai T20 TEM was used to observe the samples.

### Nanoparticle tracking analysis (NTA)

According to previous reports, the NanoSight NS 300 system (NanoSight Technology, Malvern, UK) was used to track the size and density of exosomes. The exosomes were resuspended in PBS at a concentration of 5 μg/mL and further diluted 100–500-fold to achieve 20–100 objects per frame. Samples were manually injected into the sample chamber at ambient temperature. Each sample was configured with a 488 nm laser and then measured in triplicate by a high-sensitivity sCMOS camera at camera setting 13 with an acquisition time of 30 s and a detection threshold setting of 7. At least 200 completed tracks were analyzed per video, and then NTA analytical software (version 2.3) was used to analyze the data.

### Mass spectrum analysis

In our study, LC-MS/MS analysis was performed on a Q Exactive mass spectrometer (Thermo Scientific) coupled to Easy nLC (Thermo Fisher Scientific). The mass spectrometer was used in positive ion mode, and the other mode settings were as follows: automatic gain control target was set to 3e6, maximum injection time to 10 ms, and dynamic exclusion duration to 40.0 s. The resolution of survey scans was set to 70,000 at m/z 200, and the resolution of HCD spectra was set to 17,500 at m/z 200. The isolation width of the mass spectrometer was set to 2 m/z. The normalized collision energy was 30 eV, and the underfill ratio was specified as 0.1%. The instrument was run with peptide recognition mode enabled. Finally, the MS data were acquired by choosing the most abundant precursor ions dynamically from the survey scan (300–1800 m/z) for HCD fragmentation. The MS data were analyzed by MaxQuant software version 1.5.3.17 (Max Planck Institute of Biochemistry, Martinsried, Germany) [61].

### Fatty acid (FA) quantification

The concentrations of the FAs C16:1, C18:1, C20:4, and C22:4 were quantified in cells by LC/MS as described previously [62].

### Western blotting analysis

The expression levels of proteins, including SCD1, hnRNPA1, and USP7, were validated by WB analysis, and samples were standardized to GAPDH or α-Tubulin. The membrane was blocked in 5% fat-free dry milk for 1 h and incubated overnight with the primary antibodies. Subsequently, the membrane was incubated with secondary antibody at room temperature for 1 h. The following primary antibodies were used: anti-SCD1 (Cell Signaling Technology, 2438), anti-hnRNPA1 (Santa Cruz, sc-32301), anti-TSG101 (Santa Cruz, sc-7964), anti-CD9 (Santa Cruz, sc-13118), anti-Alix (Santa Cruz, sc-53540), anti-USP7 (Santa Cruz, sc-137001), anti-NOTCH1 (Abcam, ab52627), anti-SOX9 (Abcam, ab185966), anti-OCT4 (Abcam, ab181557), anti-ZRANB2 (Santa Cruz, sc-514200), anti-SFRS9 (Proteintech, 17926-1-AP), anti-α-Tubulin (Santa Cruz, sc-8035), anti-Ubiquitin (Santa Cruz, sc-8017), and anti-GAPDH (Santa Cruz, sc-47724). Primary antibodies were detected with the corresponding secondary antibodies, goat anti-mouse IgG-HRP (Santa Cruz, sc-2005), and goat anti-rabbit IgG-HRP (Santa Cruz, sc-2004).

### Quantitative real-time PCR (qRT-PCR)

Total RNA was extracted from GC tissues or the cultured cells by using Trizol Reagent (Invitrogen) in accordance with the manufacturer’s protocol. qRT-PCR was performed as previously described [63]. The primers for SCD1, GAPDH, CASP3, PTGS2, U1 snRNA, lncFERO, NOTCH1, SOX9, and OCT4 are listed in Supplementary Table 2. GAPDH was the internal control of lncFERO, and mRNA levels were also normalized to GAPDH. A comparative CT method was used to compare the target genes to control group. Each experiment was repeated three times.

### Determination of lipid ROS levels

After different treatments, cells were stained with 10 μM C11-BODIPY^581/591^ probe (Invitrogen) for 30 min was used to detect the level of lipid ROS according to the manufacturer’s protocol. C11-BODIPY^581/591^ is a lipid-soluble ratiometric fluorescent indicator of lipid peroxidation. Upon oxidation in live cells, the reagent shifts fluorescence emission peak from 590 nm (red) to 510 nm (green) [64]. Analysis of C11-BODIPY^581/591^ fluorescence was performed by a BD Accuri C6 flow cytometer.

### CCK-8 assay

Cells were inoculated in 96-well plates. After treatment with the designate conditions, cell viability was measured using the Cell Counting Kit-8 (CCK-8, Biosharp, China) assay. Ten microliter CCK-8 reagent was added to each well and the cells were incubated further for 2.5 h at 37 °C. The optical density value was measured at 450 nm. The following formula was used to calculate the cell inhibiting rate: Cell inhibiting rate (%) = [(*Ac* − *Ae*) / (*Ac* − *Ab*)] × 100% (*Ac* = the absorbance of the control well, *Ae* = the absorbance of the experimental well, *Ab* = the absorbance of the blank well).

### Determination of cell death

A PI (Roche) assay was used to determine cell death. The procedure was as follows. First, cells were seeded in a 6-well plate and then treated with the appropriate treatment according to the experimental designs. At the time of harvest, the cells were stained with 2 μg/mL PI. Finally, a BD Accuri C6 flow cytometer (BD Biosciences) was used to analyze dead cells (PI-positive cells).

### Cell-cycle assays

Cells for cell-cycle assays were stained with PI using DNA labeling solution kit (Roche) according to the protocol, and were analyzed by a BD Accuri C6 flow cytometer (BD Biosciences). The percentages of the cells in G0-G1 and G2-S phase were counted and compared by using Modfit LT3.1 (Verity Software, Topsham, ME, USA.)

### Mitochondrial membrane potential (MMP) measurement

MMP was estimated by Tetramethylrhodamine (Invitrogen). The GCSCs, treated with the designate conditions, were stained with 200 nM TMRE for 30 min. Then, the stained cells were washed with PBS, trypsinized and resuspended in PBS plus 2% FBS. Subsequently, fluorescence at *Ex*/*Em* = 548/574 nm was analyzed using a flow cytometer.

### Immunoprecipitation

Cells were lysed in lysis buffer containing 150 mM KCl, 25 mM Tris-HCl, pH 7.4, 5 mM EDTA, 0.5% Triton X-100, 5 mM dithiothreitol, PMSF, and a cocktail. The supernatant was mixed with anti-hnRNPA1 antibody (Abcam), anti-USP7 antibody (Santa Cruz), or anti-IgG antibody (Isotype Control) at 4 °C overnight and then incubated with beads (Santa Cruz) at room temperature for 2–4 h. Finally, the samples were washed in lysis buffer and then analyzed by WB analysis.

### Biotin RNA pulldown assay

Cell lysates from GCSCs were incubated with 100 pmol synthetic single-stranded probe containing a biotin modification overnight at 4 °C. In each binding reaction, agarose beads (USA) were added and further incubated for 4 h at 4 °C. Then, the precipitates were washed five times, boiled in SDS buffer, and subjected to WB analysis. Or isolated the coprecipitated RNAs from the precipitates by using TRIzol RNA extraction reagent according to the manufacturer’s protocol. Finally, the purified RNAs were subjected to qRT-PCR analysis.

### RNA immunoprecipitation (RIP)

Added anti-hnRNPA1 antibody (5 µg) or anti-IgG antibody (Isotype Control) to beads (40–60 µL, USA) and incubated for 30 min at RT with gentle rotation. Next, cell lysates from GCSCs were added to a combination of anti-hnRNPA1 antibody and beads, and incubated to overnight at 4 °C with gentle rotation. After the precipitates were washed five times, coprecipitated RNAs were isolated by using TRIzol RNA extraction reagent according to manufacturer’s protocol. Finally, the purified RNAs were subjected to qRT-PCR analysis.

### Bioinformatics

Prediction and analysis for the lncFERO target or the interaction of lncFERO, hnRNPA1 and SCD1 mRNA were performed with the algorithms from RBPDB (http://rbpdb.ccbr.utoronto.ca/) or IntraRNA (http://rna.informatik.uni-freiburg.de/IntaRNA/).

### Establishment of tumors in nude mice

Briefly, nude mice were randomly divided into groups as described in Fig. 8A, and we were not blinded to the group allocation during the experiment. The prepared SGC7901 cells were injected subcutaneously into each mouse (5 × 10^6^ cells per mouse). The tumor-implanted mice were injected via the lateral tail vein with either cisplatin (5 μg/g) or saline every 4 days starting on day 12. The nude mice were sacrificed, tumors were removed, and serum was harvested on the 30th day.

### Statistics

All experiments were performed three times, and all quantitative data are presented as the mean value ± standard deviation. Statistical tests were performed using Student’s *t*-test in SPSS statistical software. Values of *p* < 0.05 were considered significant. * indicates *p* < 0.05; ** indicates *p* < 0.01, and *** indicates *p* < 0.001.

## Supplementary information

Supplementary Information

Supplementary Table 1

Supplementary Table 2

## References

[1] Jung E, Osswald M, Ratliff M, Dogan H, Xie R, Weil S (2021). Tumor cell plasticity, heterogeneity, and resistance in crucial microenvironmental niches in glioma. Nat Commun.

[2] Karthikeyan S, Waters IG, Dennison L, Chu D, Donaldson J, Shin DH (2021). Hierarchical tumor heterogeneity mediated by cell contact between distinct genetic subclones. J Clin Invest.

[3] Konen JM, Rodriguez BL, Padhye A, Ochieng JK, Gibson L, Diao L (2021). Dual inhibition of MEK and AXL targets tumor cell heterogeneity and prevents resistant outgrowth mediated by the epithelial-to-mesenchymal transition in NSCLC. Cancer Res.

[4] Giraud J, Molina-Castro S, Seeneevassen L, Sifré E, Izotte J, Tiffon C (2020). Verteporfin targeting YAP1/TAZ-TEAD transcriptional activity inhibits the tumorigenic properties of gastric cancer stem cells. Int J Cancer.

[5] Yang L, Shi P, Zhao G, Xu J, Peng W, Zhang J (2020). Targeting cancer stem cell pathways for cancer therapy. Signal Transduct Target Ther.

[6] Nguyen PH, Giraud J, Staedel C, Chambonnier L, Dubus P, Chevret E (2016). All-trans retinoic acid targets gastric cancer stem cells and inhibits patient-derived gastric carcinoma tumor growth. Oncogene.

[7] Courtois S, Durán RV, Giraud J, Sifré E, Izotte J, Mégraud F (2017). Metformin targets gastric cancer stem cells. Eur J Cancer.

[8] Badgley MA, Kremer DM, Maurer HC, DelGiorno KE, Lee HJ, Purohit V (2020). Cysteine depletion induces pancreatic tumor ferroptosis in mice. Science.

[9] Battaglia AM, Chirillo R, Aversa I, Sacco A, Costanzo F, Biamonte F (2020). Ferroptosis and cancer: mitochondria meet the “Iron Maiden” cell death. Cells.

[10] Jiang L, Kon N, Li T, Wang SJ, Su T, Hibshoosh H (2015). Ferroptosis as a p53-mediated activity during tumour suppression. Nature.

[11] Lee J, You JH, Shin D, Roh JL (2020). Inhibition of glutaredoxin 5 predisposes cisplatin-resistant head and neck cancer cells to ferroptosis. Theranostics.

[12] Guo J, Xu B, Han Q, Zhou H, Xia Y, Gong C (2018). Ferroptosis: a novel anti-tumor action for cisplatin. Cancer Res Treat.

[13] Wang Y, Yu L, Ding J, Chen Y (2018). Iron metabolism in cancer. Int J Mol Sci.

[14] Kirtonia A, Sethi G, Garg M (2020). The multifaceted role of reactive oxygen species in tumorigenesis. Cell Mol life Sci.

[15] Yuan H, Pratte J, Giardina C (2021). Ferroptosis and its potential as a therapeutic target. Biochemical Pharmacol.

[16] Su Y, Zhao B, Zhou L, Zhang Z, Shen Y, Lv H (2020). Ferroptosis, a novel pharmacological mechanism of anti-cancer drugs. Cancer Lett.

[17] Snaebjornsson MT, Janaki-Raman S, Schulze A (2020). Greasing the wheels of the cancer machine: the role of lipid metabolism in cancer. Cell Metab.

[18] Rysman E, Brusselmans K, Scheys K, Timmermans L, Derua R, Munck S (2010). De novo lipogenesis protects cancer cells from free radicals and chemotherapeutics by promoting membrane lipid saturation. Cancer Res.

[19] Kopecka J, Trouillas P, Gašparović A, Gazzano E, Assaraf YG, Riganti C (2020). Phospholipids and cholesterol: inducers of cancer multidrug resistance and therapeutic targets. Drug Resist Updat.

[20] Jiang X, Stockwell BR, Conrad M (2021). Ferroptosis: mechanisms, biology and role in disease. Nat Rev Mol Cell Biol.

[21] Gao Y, Li J, Xi H, Cui J, Zhang K, Zhang J (2020). Stearoyl-CoA-desaturase-1 regulates gastric cancer stem-like properties and promotes tumour metastasis via Hippo/YAP pathway. Br J Cancer.

[22] Pérez-Heras AM, Mayneris-Perxachs J, Cofán M, Serra-Mir M, Castellote AI, López-Sabater C (2018). Long-chain n-3 PUFA supplied by the usual diet decrease plasma stearoyl-CoA desaturase index in non-hypertriglyceridemic older adults at high vascular risk. Clin Nutr.

[23] Bednarski T, Olichwier A, Opasinska A, Pyrkowska A, Gan AM, Ntambi JM (2016). Stearoyl-CoA desaturase 1 deficiency reduces lipid accumulation in the heart by activating lipolysis independently of peroxisome proliferator-activated receptor α. Biochim Biophys Acta.

[24] Tesfay L, Paul BT, Konstorum A, Deng Z, Cox AO, Lee J (2019). Stearoyl-CoA desaturase 1 protects ovarian cancer cells from ferroptotic cell death. Cancer Res.

[25] Luis G, Godfroid A, Nishiumi S, Cimino J, Blacher S, Maquoi E (2021). Tumor resistance to ferroptosis driven by Stearoyl-CoA Desaturase-1 (SCD1) in cancer cells and Fatty Acid Biding Protein-4 (FABP4) in tumor microenvironment promote tumor recurrence. Redox Biol.

[26] Kagan VE, Mao G, Qu F, Angeli JP, Doll S, Croix CS (2017). Oxidized arachidonic and adrenic PEs navigate cells to ferroptosis. Nat Chem Biol.

[27] Yang WS, Kim KJ, Gaschler MM, Patel M, Shchepinov MS, Stockwell BR (2016). Peroxidation of polyunsaturated fatty acids by lipoxygenases drives ferroptosis. Proc Natl Acad Sci USA.

[28] Théry C, Zitvogel L, Amigorena S (2002). Exosomes: composition, biogenesis and function. Nat Rev Immunol.

[29] Zhang H, Deng T, Ge S, Liu Y, Bai M, Zhu K (2019). Exosome circRNA secreted from adipocytes promotes the growth of hepatocellular carcinoma by targeting deubiquitination-related USP7. Oncogene.

[30] Zhang H, Deng T, Liu R, Bai M, Zhou L, Wang X (2017). Exosome-delivered EGFR regulates liver microenvironment to promote gastric cancer liver metastasis. Nat Commun.

[31] Cooks T, Pateras IS, Jenkins LM, Patel KM, Robles AI, Morris J (2018). Mutant p53 cancers reprogram macrophages to tumor supporting macrophages via exosomal miR-1246. Nat Commun.

[32] Garg M, Sethi G (2021). Emerging role of long non-coding RNA (lncRNA) in human malignancies: A unique opportunity for precision medicine. Cancer Lett.

[33] Yousefi H, Maheronnaghsh M, Molaei F, Mashouri L, Reza Aref A, Momeny M (2020). Long noncoding RNAs and exosomal lncRNAs: classification, and mechanisms in breast cancer metastasis and drug resistance. Oncogene.

[34] Xu YH, Deng JL, Wang G, Zhu YS (2019). Long non-coding RNAs in prostate cancer: Functional roles and clinical implications. Cancer Lett.

[35] Tan H, Zhang S, Zhang J, Zhu L, Chen Y, Yang H (2020). Long non-coding RNAs in gastric cancer: New emerging biological functions and therapeutic implications. Theranostics.

[36] Xu Y, Qiu M, Shen M, Dong S, Ye G, Shi X (2021). The emerging regulatory roles of long non-coding RNAs implicated in cancer metabolism. Mol Ther.

[37] Mao C, Wang X, Liu Y, Wang M, Yan B, Jiang Y (2018). A G3BP1-interacting lncRNA promotes ferroptosis and apoptosis in cancer via nuclear sequestration of p53. Cancer Res.

[38] Qi W, Li Z, Xia L, Dai J, Zhang Q, Wu C (2019). LncRNA GABPB1-AS1 and GABPB1 regulate oxidative stress during erastin-induced ferroptosis in HepG2 hepatocellular carcinoma cells. Sci Rep.

[39] Yang Y, Tai W, Lu N, Li T, Liu Y, Wu W (2020). lncRNA ZFAS1 promotes lung fibroblast-to-myofibroblast transition and ferroptosis via functioning as a ceRNA through miR-150-5p/SLC38A1 axis. Aging.

[40] Igal RA (2016). Stearoyl CoA desaturase-1: new insights into a central regulator of cancer metabolism. Biochim Biophys Acta.

[41] Magtanong L, Ko PJ, Dixon SJ (2016). Emerging roles for lipids in non-apoptotic cell death. Cell Death Differ.

[42] Martens-Uzunova ES, Böttcher R, Croce CM, Jenster G, Visakorpi T, Calin GA (2014). Long noncoding RNA in prostate, bladder, and kidney cancer. Eur Urol.

[43] Ren X, Chen C, Luo Y, Liu M, Li Y, Zheng S (2020). lncRNA-PLACT1 sustains activation of NF-κB pathway through a positive feedback loop with IκBα/E2F1 axis in pancreatic cancer. Mol Cancer.

[44] Clarke MF (2019). Clinical and therapeutic implications of cancer stem cells. N Engl J Med.

[45] Fukuda K, Saikawa Y, Ohashi M, Kumagai K, Kitajima M, Okano H (2009). Tumor initiating potential of side population cells in human gastric cancer. Int J Oncol.

[46] Takaishi S, Okumura T, Tu S, Wang SS, Shibata W, Vigneshwaran R (2009). Identification of gastric cancer stem cells using the cell surface marker CD44. Stem Cells.

[47] Ye Z, Zhuo Q, Hu Q, Xu X, Mengqi L, Zhang Z (2021). FBW7-NRA41-SCD1 axis synchronously regulates apoptosis and ferroptosis in pancreatic cancer cells. Redox Biol.

[48] Wu B, Su S, Patil DP, Liu H, Gan J, Jaffrey SR (2018). Molecular basis for the specific and multivariant recognitions of RNA substrates by human hnRNP A2/B1. Nat Commun.

[49] Qin X, Guo H, Wang X, Zhu X, Yan M, Wang X (2019). Exosomal miR-196a derived from cancer-associated fibroblasts confers cisplatin resistance in head and neck cancer through targeting CDKN1B and ING5. Genome Biol.

[50] López de Andrés J, Griñán-Lisón C, Jiménez G, Marchal JA (2020). Cancer stem cell secretome in the tumor microenvironment: a key point for an effective personalized cancer treatment. J Hematol Oncol.

[51] Shen M, Dong C, Ruan X, Yan W, Cao M, Pizzo D (2019). Chemotherapy-induced extracellular vesicle miRNAs promote breast cancer stemness by targeting ONECUT2. Cancer Res.

[52] Chen X, Kang R, Kroemer G, Tang D (2021). Broadening horizons: the role of ferroptosis in cancer. Nat Rev Clin Oncol.

[53] Wu Y, Zhang S, Gong X, Tam S, Xiao D, Liu S (2020). The epigenetic regulators and metabolic changes in ferroptosis-associated cancer progression. Mol Cancer.

[54] Gnanapradeepan K, Basu S, Barnoud T, Budina-Kolomets A, Kung CP, Murphy ME (2018). The p53 tumor suppressor in the control of metabolism and ferroptosis. Front Endocrinol.

[55] Yang WS, SriRamaratnam R, Welsch ME, Shimada K, Skouta R, Viswanathan VS (2014). Regulation of ferroptotic cancer cell death by GPX4. Cell.

[56] Dolma S, Lessnick SL, Hahn WC, Stockwell BR (2003). Identification of genotype-selective antitumor agents using synthetic lethal chemical screening in engineered human tumor cells. Cancer Cell.

[57] Yagoda N, von Rechenberg M, Zaganjor E, Bauer AJ, Yang WS, Fridman DJ (2007). RAS-RAF-MEK-dependent oxidative cell death involving voltage-dependent anion channels. Nature.

[58] Silva MM, Rocha CRR, Kinker GS, Pelegrini AL, Menck CFM (2019). The balance between NRF2/GSH antioxidant mediated pathway and DNA repair modulates cisplatin resistance in lung cancer cells. Sci Rep.

[59] Yang T, Shu X, Zhang HW, Sun LX, Yu L, Liu J (2020). Enolase 1 regulates stem cell-like properties in gastric cancer cells by stimulating glycolysis. Cell Death Dis.

[60] Valadi H, Ekström K, Bossios A, Sjöstrand M, Lee JJ, Lötvall JO (2007). Exosome-mediated transfer of mRNAs and microRNAs is a novel mechanism of genetic exchange between cells. Nat Cell Biol.

[61] Cox J, Mann M (2008). MaxQuant enables high peptide identification rates, individualized p.p.b.-range mass accuracies and proteome-wide protein quantification. Nat Biotechnol.

[62] Yao CH, Fowle-Grider R, Mahieu NG, Liu GY, Chen YJ, Wang R (2016). Exogenous fatty acids are the preferred source of membrane lipids in proliferating fibroblasts. Cell Chem Biol.

[63] Schmittgen TD, Lee EJ, Jiang J, Sarkar A, Yang L, Elton TS (2008). Real-time PCR quantification of precursor and mature microRNA. Methods.

[64] Martinez AM, Kim A, Yang WS (2020). Detection of ferroptosis by BODIPY™ 581/591 C11. Methods Mol Biol.

