# Supplementary material for: Chemotoxicity-induced exosomal lncFERO regulates ferroptosis and stemness in gastric cancer stem cells
Source: Cell Death Dis. 2021 Nov 29;12(12):1116. doi: 10.1038/s41419-021-04406-z (PMC8629982; doi:10.1038/s41419-021-04406-z)
Supplement: Supplementary file 1 — Supplementary Information [file 41419_2021_4406_MOESM1_ESM.docx]

**Supplementary Information**

**Supplementary Figures**


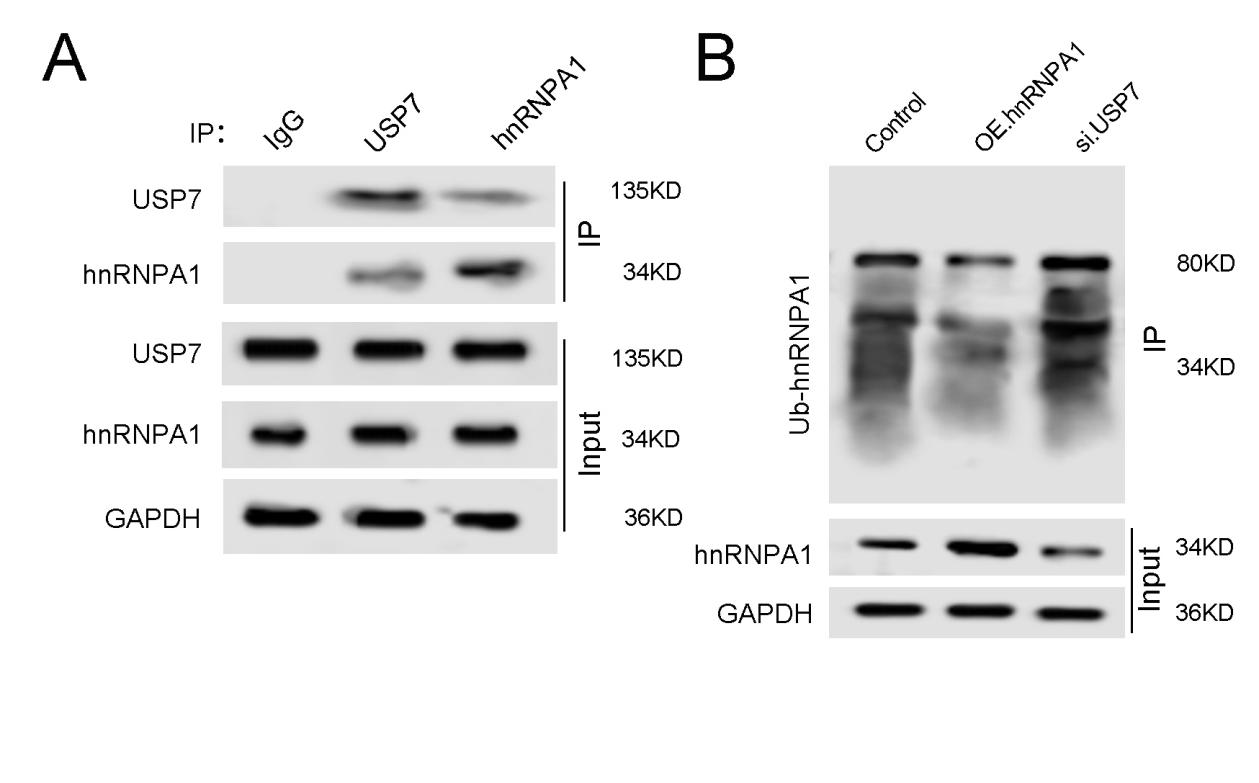


**Supplemental Figure 1. USP7 stabilized hnRNPA1 in GC cells via deubiquitination.**

A. Detection of USP7 and hnRNPA1 by using immuno-precipitation assay. B. The relationship between USP7 and the levels of hnRNA1 ubiquitination.


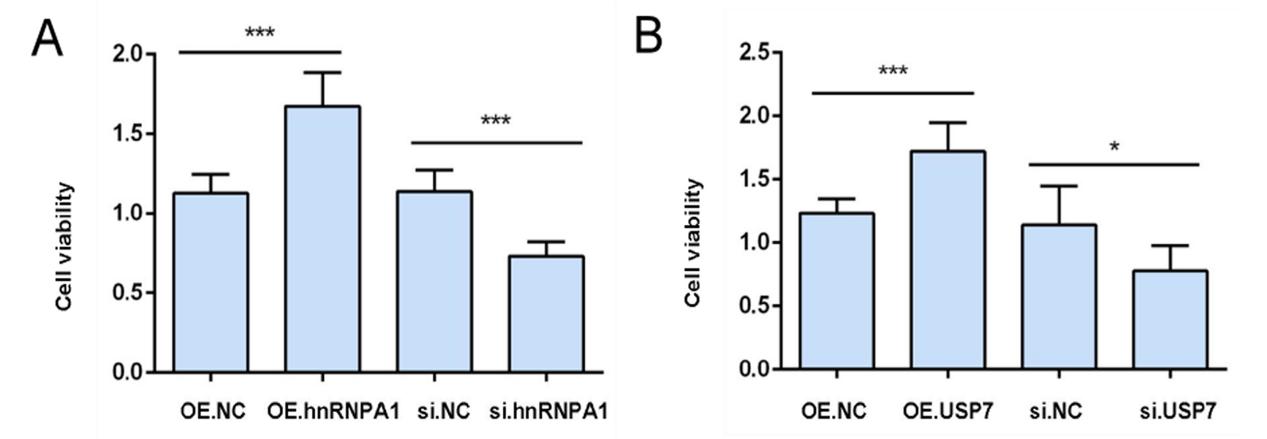


**Supplemental Figure 2. The effect of hnRNPA1 and USP7 on cell growth in GC cells.**

A. Detection of the cell viability by Cell Counting Kit-8 in OE.NC, OE.hnRNPA1, si.NC and si.hnRNPA1 GC cells. B. Detection of the cell viability by Cell Counting Kit-8 in OE.NC, OE.USP7, si.NC and si.USP7 GC cells


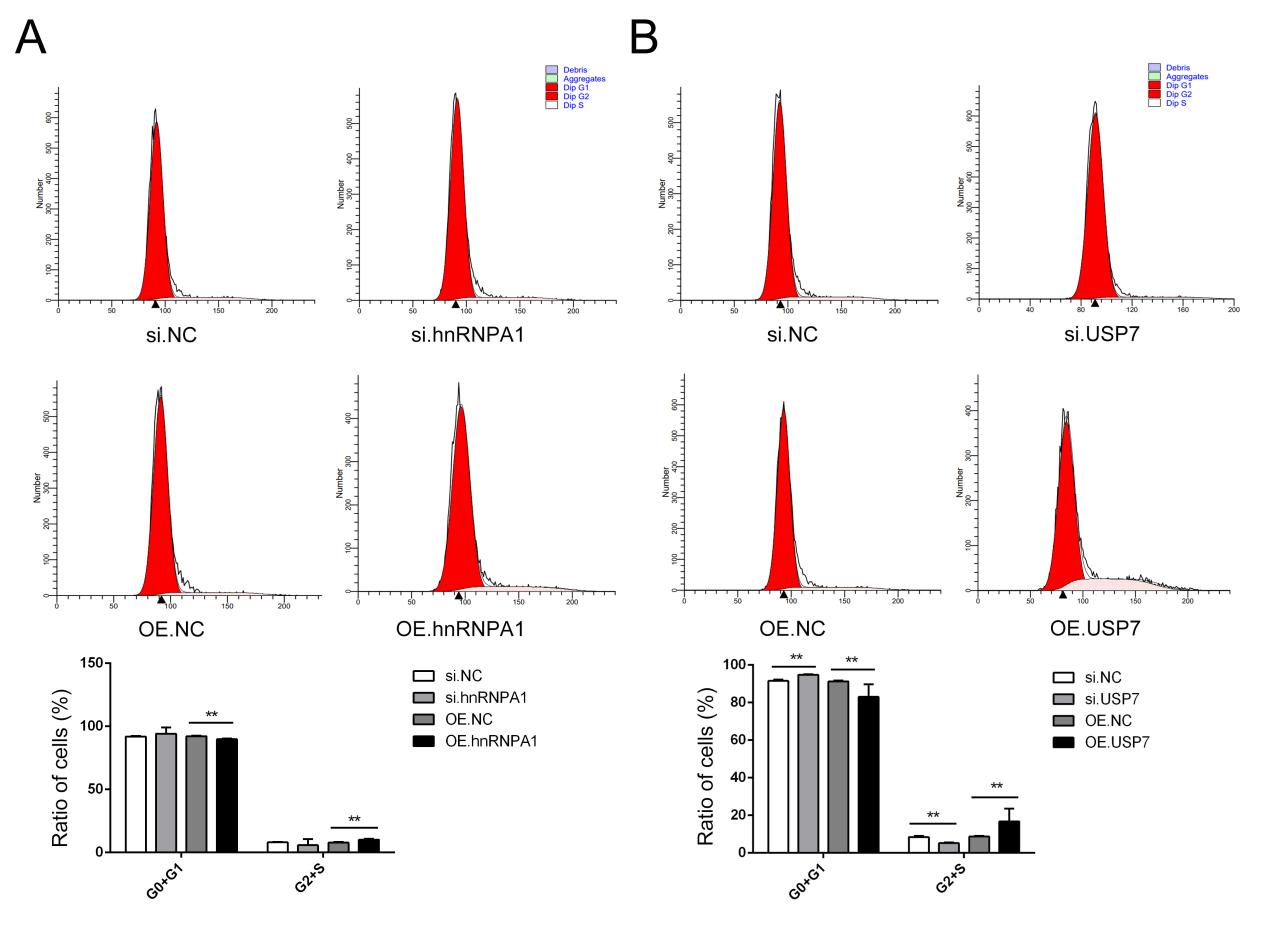


**Supplemental Figure 3. The effect of hnRNPA1 and USP7 on cell cycle in GC cells.**

A. Detection of the numbers of cells in different cell cycles by flowcytometry in si.NC, si.hnRNPA1, OE.NC and OE.hnRNPA1 GC cells. B. Detection of the numbers of cells in different cell cycles by flowcytometry in si.NC, si.USP7, OE.NC and OE.USP7 GC cells.


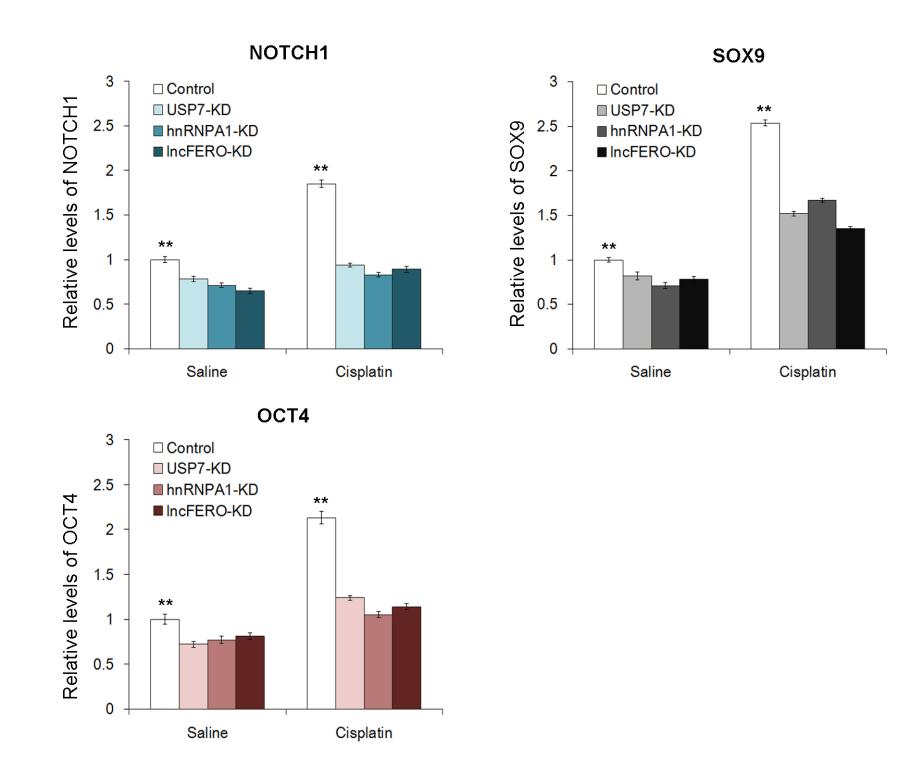


**Supplemental Figure 4. Detection of stemness-associated genes level in vivo.** NOTCH1, SOX9 and OCT4 were inhibited in the USP7-KD, hnRNPA1-KD and lncFERO-KD groups, and cisplatin improved the inhibition relatively.


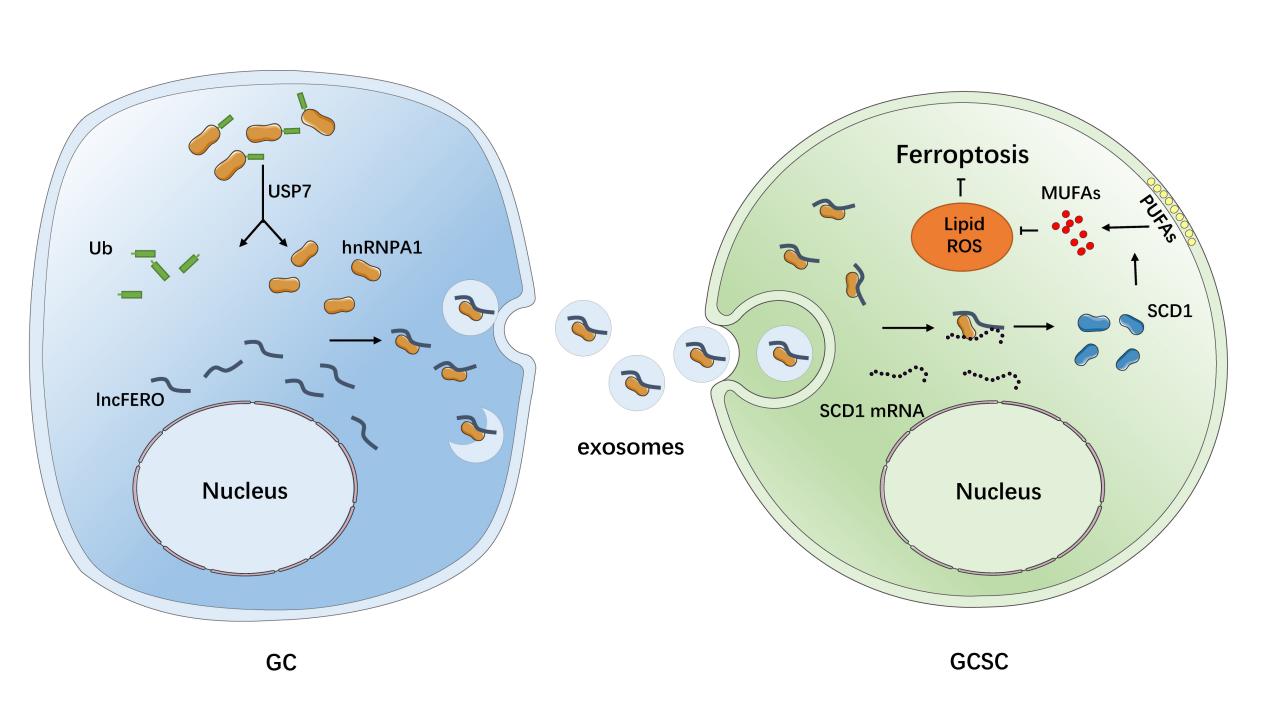


**Supplemental Figure 5. A model illustrating the role of GC-derived exosomal lncFERO in regulating ferroptosis in GCSC.**

**Supplementary Tables**

**Supplemental Table 1. Top 20 up-regulated lncRNAs in the serum exosomes of patients with gastric cancer compared with those in controls.** From: [Microarray analysis of lncRNA expression profiles in](https://lipidworld.biomedcentral.com/articles/10.1186/s12944-020-01348-x) the serum exosomes of patients with gastric cancer.

**Supplemental Table 2. Primer sequences of releated genes for qRT-PCR.**
